# Performance of sFlt-1/PIGF Ratio for the Prediction of Perinatal Outcome in Obese Pre-Eclamptic Women

**DOI:** 10.3390/jcm11113023

**Published:** 2022-05-27

**Authors:** Anne Karge, Linus Desing, Bernhard Haller, Javier U. Ortiz, Silvia M. Lobmaier, Bettina Kuschel, Oliver Graupner

**Affiliations:** 1 Department of Obstetrics and Gynecology, University Hospital Rechts der Isar, Technical University of Munich, 80333 Munich, Germany; linus.desing@mri.tum.de (L.D.); javier.ortiz@mri.tum.de (J.U.O.); silvia.lobmaier@mri.tum.de (S.M.L.); bettina.kuschel@mri.tum.de (B.K.); ograupner@ukaachen.de (O.G.); 2 Institute of AI and Informatics in Medicine, University Hospital Rechts der Isar, Technical University of Munich, 80333 Munich, Germany; bernhard.haller@mri.tum.de; 3 Department of Obstetrics and Gynecology, University Hospital Aachen, RWTH University, 52062 Aachen, Germany

**Keywords:** preeclampsia, (anti-)angiogenic factors, placental growth factor, soluble fms-like tyrosine kinase 1, obesity

## Abstract

Obese women are at high risk of developing pre-eclampsia (PE). As an altered angiogenic profile is characteristic for PE, measurement of soluble fms-like tyrosine kinase-1 (sFlt-1)/placental growth factor (PIGF) ratio in the maternal serum can be helpful for PE diagnosis, as well as for adverse perinatal outcome (APO) prediction. There is growing evidence that obesity might influence the level of sFlt-1/PIGF and, therefore, the aim of the study was the evaluation of sFlt-1/PIGF as an APO predictor in obese women with PE. Pre-eclamptic women who had an sFlt-1/PIGF measurement at the time of diagnosis were retrospectively included. Women were classified according to their pre-pregnancy body mass index (BMI) as normal weight (BMI < 25 kg/m^2^), overweight (BMI > 25–29.9 kg/m^2^) or obese (BMI ≥ 30 kg/m^2^). APO was defined as the occurrence of one of the following outcomes: Small for gestational age, defined as a birthweight < 3rd centile, neonatal mortality, neonatal seizures, admission to neonatal unit required (NICU) or respiratory support. A total of 141 women were included. Of them, 28 (20%) patients were obese. ROC (receiver operating characteristic) analysis revealed a high predictive value for sFlt-1/PIGF and APO across the whole study cohort (AUC = 0.880, 95% CI: 0.826–0.936; *p* < 0.001). However, the subgroup of obese women showed a significantly lower level of sFlt-1 and, therefore, the performance of sFlt-1/PIGF as APO predictor was poorer compared to normal or overweight PE women (AUC = 0.754, 95% CI: 0.552–0.956, *p* = 0.025). In contrast to normal or overweight women, a ratio of sFlt-1/PIGF < 38 could not rule out APO in women with obesity.

## 1. Introduction

Pre-eclampsia (PE) is a disease affecting 2–8% of all pregnancies and a major cause for fetal and maternal morbidity [1,2,3]. An altered inflammatory and angiogenic profile is a main finding in the pathophysiology of PE and serum markers, such as soluble fms-like tyrosine kinase-1 (sFlt-1) and placental growth factor (PIGF), are now established for the diagnosis of PE [4,5].

Around a third of pregnant women in Western countries are affected by overweight and the percentage is steadily increasing [6,7]. In 2017, 15% of pregnant women in Germany were obese compared to 12% in 2003 [8]. Obesity is a risk factor for the development of PE [9] and is, as well as PE, characterized by an endothelial dysfunction and a proinflammatory microenvironment [10]. Therefore, the evaluation of the established predictive marker of PE, sFlt-1/PIGF ratio, is of the utmost importance in this high-risk cohort. Recent guidelines recommend the use of sFlt-1/PIGF for diagnosis or exclusion of PE [11]. The PROGNOSIS study, which evaluated and established sFlt-1/PIGF as a diagnostic tool, did not differentiate between obese or normal-weight women and did not, therefore, examine if different cutoffs are necessary or not [12].

The ratio of sFlt-1/PIGF is not only used for diagnosing or excluding PE, but also for the prediction of adverse perinatal outcome (APO) and adverse maternal outcome (AMO) [13,14,15,16]. Specifically, highly elevated levels of sFlt-1/PIGF correlate with APO and might be helpful for estimating the mean time until delivery [12,17,18,19,20]. Likewise, published data do not discriminate between different BMI groups.

It is important to notice that not only the severity of PE itself, but also other factors influence the serum level of sFlt-1/PIGF, such as twin pregnancies or, as mentioned above, BMI [21,22]. Levels of sFlt-1 seem to be inversely correlated with BMI and, therefore, APO prediction by sFlt-1/PIGF might be distorted [23].

The aim of this study was the evaluation of sFlt-1/PIGF as an APO and AMO predictor in obese women with PE and/or HELLP syndrome.

## 2. Materials and Methods

This is a retrospective single-center study from January 2018 to December 2020 at the University Hospital rechts der Isar, Department of Obstetrics and Gynecology (Technical University of Munich). All patients with diagnosed PE and/or HELLP syndrome were included if sFlt-1/PIGF was determined at the time of diagnosis. Cases with missing data on perinatal outcome as well as multiple pregnancies were excluded. Additionally, we included the study cohort of women with late-onset PE which was described before [24].

Elevated blood pressure was defined as a new onset hypertension with a systolic blood pressure of 140 mmHg and/or a diastolic blood pressure of 90 mmHg, on two occasions at least 4 h apart [25]. Large cuffs were used for obese women.

PE was defined as elevated blood pressure and one of the following symptoms: proteinuria (≥300 mg/24 h), thrombocytopenia (platelet count less than 100.000/microliter), poor liver function (elevated blood levels of liver transaminases to twice the normal concentration), new-onset renal insufficiency (elevated serum creatinine greater than 1.1 mg/dL or a doubling of serum creatinine in the absence of other renal disease), pulmonary edema or new-onset cerebral or visual disturbances [26]. HELLP syndrome was defined as the occurrence of hemolysis (haptoglobin < 10 mg/L), elevated liver enzymes (twice the normal concentration) and low platelets (<100.000/microliter) [27].

Diagnosis of PE < 34 weeks was classified as early onset (eo) PE and diagnosis of PE ≥ 34 weeks as late-onset (lo) PE [28].

APO was defined as the presence of at least one of the following outcomes according to the Delphi consensus defining the core outcome of PE [29]: Small for gestational age defined as a birthweight < 3rd centile according to local standards [30], neonatal mortality, neonatal seizures, admission to neonatal unit required (NICU) or respiratory support. There was no case of stillbirth.

Adverse maternal outcome (AMO) was defined as the occurrence of at least one of the following core outcomes of PE: eclampsia, pulmonary edema, acute kidney injury (defined as elevated serum creatinine greater than 1.1 mg/dL or a doubling of serum creatinine in the absence of other renal disease), placental abruption, HELLP syndrome, admission to intensive care unit or the need of intubation and mechanical ventilation. There was no case of maternal mortality, cortical blindness, retinal detachment, liver capsule hematoma or rupture or stroke.

The sFlt-1/PIGF ratio was determined at the time of diagnosis according to our local chemistry guidelines as previously described [18].

We used the cutoff values for sFlt-1/PlGF, which are recommended by the consensus statement [31]: <38 exclusion of PE (gestational-age independent) for at least 1 week, >85 diagnosis of early onset (eo) PE (<34 week of gestation) and >110 diagnosis of late-onset (lo) PE (≥34 week of gestation).

Overweight and obesity were defined according to the WHO guidelines as a BMI from 25 to 29.9 kg/m^2^ or >30 kg/m^2^, respectively [32]. A BMI <25 kg/m^2^ was considered as normal weight. The pre-pregnancy BMI was used for classification.

### 2.1. Statistical Analysis

We used IBM SPSS Statistics for Windows, version 28 (IBM Corp., Armonk, NY, USA) and R version 4.1.2 (R Foundation for Statistical Computing, Vienna, Austria) for our statistical analysis. Quantitative data are shown as means and standard deviations or median and interquartile range; categorical data are presented as absolute and relative frequencies. Differences in distributions of quantitative data between the three weight groups were tested using the Kruskal–Wallis test. If a significant difference was found pairwise group comparisons were performed with Mann–Whitney U tests. Categorical data were compared between groups using Fisher’s exact test. The predictive value of sFlt-1/PIGF for APO or AMO was analyzed with receiver operating characteristic (ROC) curves. Areas under the ROC curves were compared between groups using the method proposed by Delong et al. [33]. All statistical tests were conducted two sided and a *p*-value < 0.05 was considered statistically significant.

### 2.2. Ethical Approval

The study was approved by our local Institutional Ethics Board (Ethikkommission der Fakultät für Medizin der Technischen Universität München, protocol number 232/17). The study was not registered in a public trial registry.

## 3. Results

### 3.1. Baseline Characteristics and Perinatal Outcome

In total, 141 pregnancies with PE and/or HELLP were enrolled. Of these cases, 45 (32%) were diagnosed with early onset and 96 (68%) with late-onset PE and/or HELLP syndrome. Further, 84 women in our cohort had a normal pre-conceptional BMI (60%), 29 were overweight (20%) and 28 were obese (20%). Of all newborns, APO occurred in 69 cases (49%). Many newborns were affected by more than one event. The most common APO was admission to NICU in 55 cases (40%), followed by respiratory support in 42 cases (30%). There were 13 cases of newborns with a birthweight <3rd centile (9%), three cases of seizures (2%) and one case of neonatal death (0.7%).

AMO was observed in 36 cases (26%). The most common event was acute kidney injury in 15 cases (11%), followed by early onset HELLP syndrome affecting 10 pregnancies and late-onset HELLP syndrome affecting nine pregnancies (7% and 6%, respectively). Other AMO events were rarely observed.

Baseline characteristics of the study cohort and data on perinatal outcome are described in Table 1 and Table 2.

### 3.2. Levels of Angiogenic Factors in Preeclamptic Women Depending on BMI

**Figure 1:**
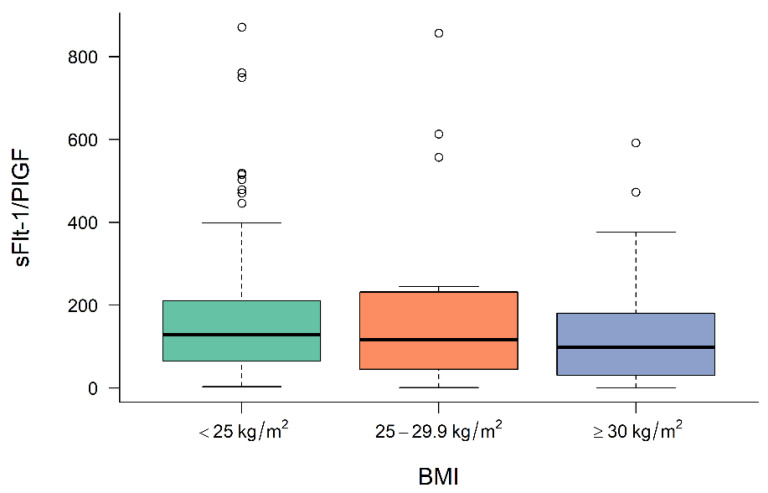
Level of sFlt-1/PIGF at diagnosis in normal-weight, overweight and obese women. BMI body mass index, sFlt-1 soluble fms-like tyrosine kinase-1, PIGF placental growth factor.

**Figure 2:**
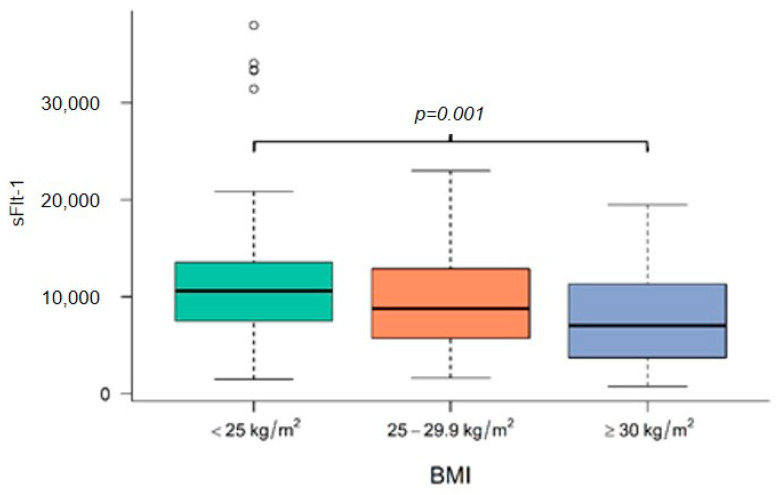
Level of sFlt-1 at diagnosis in normal-weight, overweight and obese women. BMI body mass index, sFlt-1 soluble fms-like tyrosine kinase-1.

**Figure 3:**
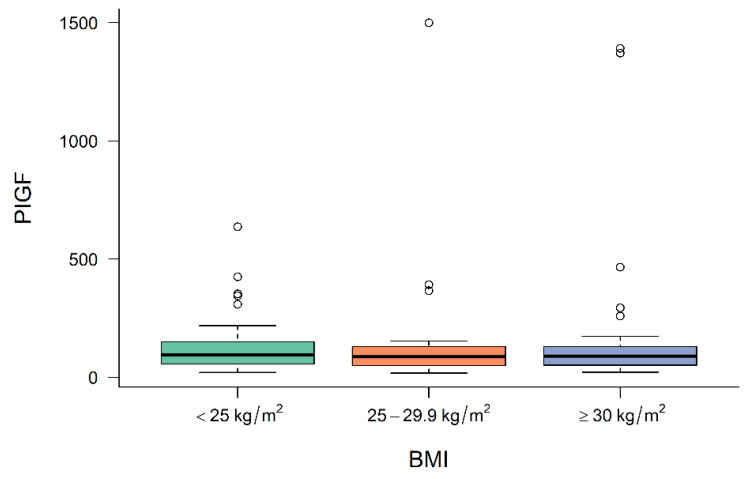
Level of PIGF at diagnosis in normal-weight, overweight and obese women. BMI body mass index, PIGF placental growth factor.

Figure 1, Figure 2 and Figure 3 demonstrate the median level of sFlt-1/PIGF, sFlt-1 and PIGF in the different BMI subgroups. Obese women show a significantly lower sFlt-1 compared to normal-weight or overweight women (*p* = 0.001), whereas sFlt-1/PIGF and PIGF are not statistically different in the different subgroups.

### 3.3. APO Prediction by sFlt-1/PlGF

For the whole study cohort, ROC analysis revealed a significant predictive value for sFlt-1/PIGF and APO (AUC = 0.880, 95% CI: 0.824–0.936; *p* < 0.001), as well as for PIGF or sFlt-1 alone (AUC = 0.866, 95% CI: 0.807–0.925, *p* < 0.001 and AUC = 0.721, 95% CI: 0.637–0.804, *p* < 0.001, respectively). The area under the ROC curve (AUC) was significantly larger for sFlt-1/PIGF and for PIGF than for sFlt-1 alone (*p* < 0.001 and *p* = 0.005).

In all the subgroups of normal weight, overweight and obese women, sFlt-1/PIGF was significantly associated with APO, although the prognostic value was reduced in obese women compared to the other two subgroups (AUC = 0.914, 95% CI: 0.857–0.972, *p* < 0.001; AUC = 0.931, 95% CI: 0.845–0.999, *p* < 0.001; AUC = 0.754, 95% CI: 0.552–0.956, *p* = 0.025). In contrast, sFlt-1 failed to be statistically significant as an outcome predictor in obese women (AUC = 0.642, 95% CI: 0.428–0.855, *p* = 0.213).

**Figure 4:**
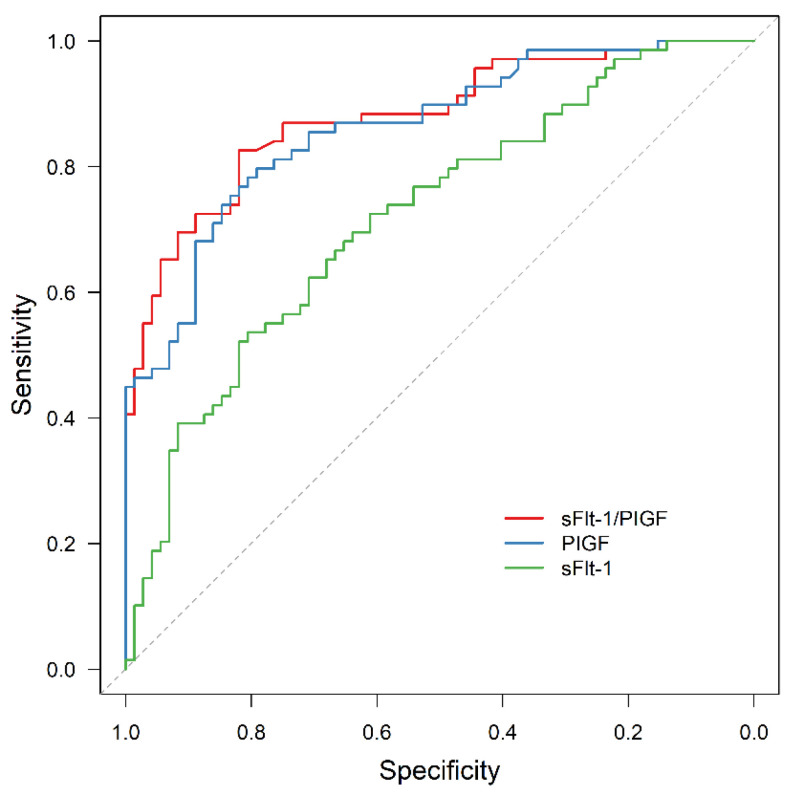
ROC curve for APO prediction by sFlt-1 (AUC = 0.721, 95% CI: 0.637–0.804, *p* < 0.001), PIGF (AUC = 0.866, 95% CI: 0.807–0.925, *p* < 0.001) and sFlt-1/PIGF (AUC = 0.880, 95% CI: 0.824–0.936, *p* < 0.001). ROC receiver operating characteristic, APO adverse perinatal outcome, sFlt-1 soluble fms-like tyrosine kinase-1, PIGF placental growth factor.

**Figure 5:**
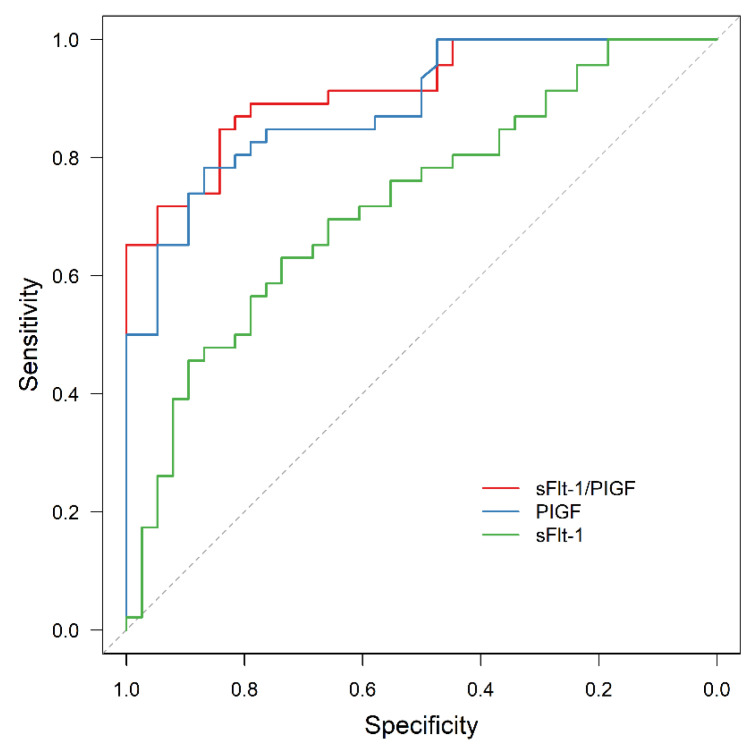
ROC curve for APO prediction in the subgroup of normal-weight women by sFlt-1 (AUC = 0.723, 95% CI: 0.615–0.832, *p* < 0.001), PIGF (AUC = 0.892, 95% CI: 0.826–0.958, *p* < 0.001) and sFlt-1/PIGF (AUC = 0.914, 95% CI: 0.857–0.972, *p* < 0.001). ROC receiver operating characteristic, APO adverse perinatal outcome, sFlt-1 soluble fms-like tyrosine kinase-1, PIGF placental growth factor.

**Figure 6:**
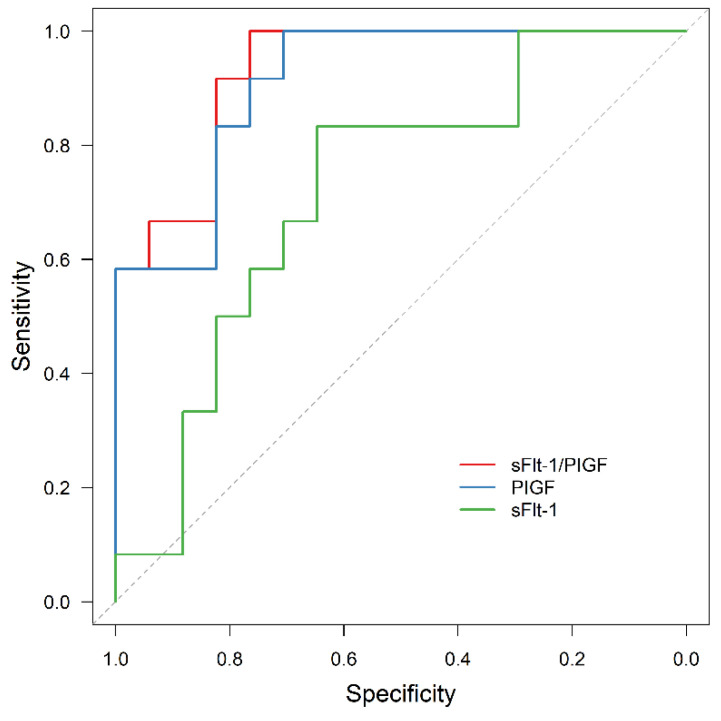
ROC curve for APO prediction in the subgroup of overweight women by sFlt-1 (AUC = 0.721, 95% CI: 0.531–0.910, *p* = 0.046), PIGF (AUC = 0.912, 95% CI: 0.811–1.000, *p* < 0.001) and sFlt-1/PIGF (AUC = 0.931, 95% CI: 0.845–0.999, *p* < 0.001). ROC receiver operating characteristic, APO adverse perinatal outcome, sFlt-1 soluble fms-like tyrosine kinase-1, PIGF placental growth factor.

**Figure 7:**
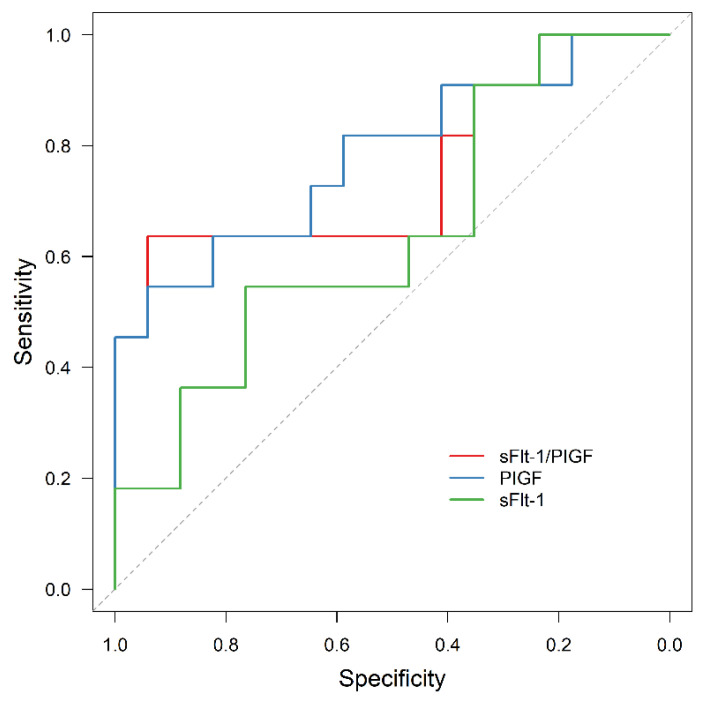
ROC curve for APO prediction in the subgroup of obese women by sFlt-1 (AUC = 0.642, 95% CI: 0.428–0.855, *p* = 0.213), PIGF (AUC = 0.781, 95% CI: 0.596–0.966, *p* = 0.014) and sFlt-1/PIGF (AUC = 0.754, 95% CI: 0.552–0.956, *p* = 0.025). ROC receiver operating characteristic, APO adverse perinatal outcome, sFlt-1 soluble fms-like tyrosine kinase-1, PIGF placental growth factor.

The results are presented in Figure 4, Figure 5, Figure 6 and Figure 7.

### 3.4. AMO Prediction by sFlt-1/PIGF

For the whole study cohort, ROC analysis revealed a predictive value for sFlt-1/PIGF and AMO (AUC = 0.667, 95% CI: 0.566–0.768; *p* = 0.003), as well as for sFlt-1 (AUC = 0.694, 95% CI: 0.598–0.790; *p* = 0.001), but not for PIGF (AUC = 0.605, 95% CI: 0.495–0.715, *p* = 0.061). Due to the small number of events, we did not perform a subgroup analysis.

**Figure 8:**
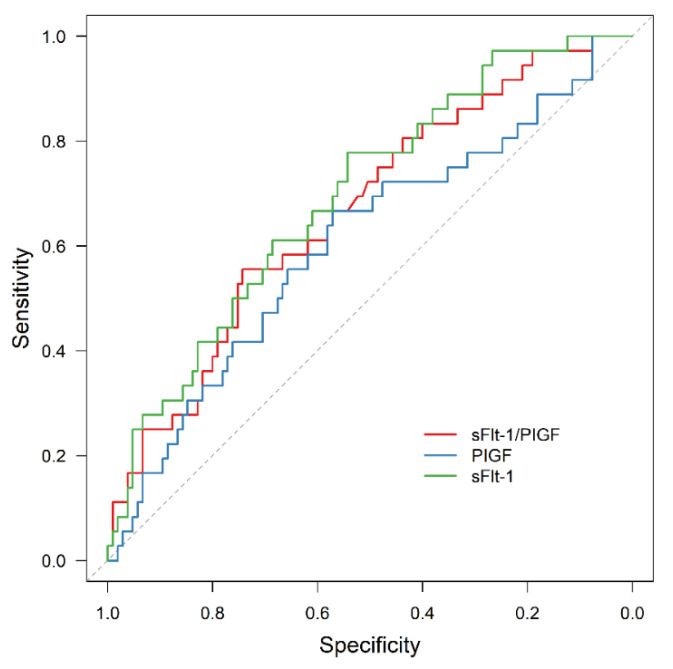
ROC curve for AMO prediction by sFlt-1 (AUC = 0.694, 95% CI: 0.598–0.790, *p* = 0.001), PIGF (AUC = 0.605, 95% CI: 0.495–0.715, *p* = 0.061) and sFlt-1/PIGF (AUC = 0.667, 95% CI: 0.566–0.768, *p* = 0.003). ROC receiver operating characteristic, APO adverse perinatal outcome, sFlt-1 soluble fms-like tyrosine kinase-1, PIGF placental growth factor.

ROC analysis is shown in Figure 8.

### 3.5. Exclusion of APO by sFlt-1/PIGF

In the group of normal-weight women, 8 of 84 (10%) women showed an sFlt-1/PIGF level < 38 and in the overweight cohort, 5 of 29 (17%) women. None of these women were affected by APO. In contrast, 7 of 28 (25%) obese women showed an sFlt-1/PIGF level < 38, but two of them were affected by APO. Both women developed superimposed PE and high blood pressure despite their antihypertensive medications, which led to iatrogenic preterm delivery with admission of the newborn to NICU.

## 4. Discussion

This study confirmed that obese women with PE show significantly lower levels of sFlt 1 compared to normal or overweight women. However, APO prediction by sFlt-1/PIGF is still possible, although with a poorer prognostic value compared to normal or overweight women.

As obesity is an epidemic disease affecting more and more pregnancies, outcome prediction in this subgroup is of urgent need. Outcome prediction in pre-eclamptic women using (anti-)angiogenic factors, especially sFlt-1/PIGF, was evaluated over the last decade [16,19,34]. In our study cohort, sFlt-1/PIGF had a high prognostic value for APO, a finding in line with previous publications of our working group [20,24]. Saleh et al. recently suggested a model using sFlt-1/PIGF, proteinuria and gestational age for outcome prediction in pregnancies diagnosed with PE [35]. Patients with normal sFlt-1/PIGF were affected by APO in less than 5%. However, in several studies APO prediction by sFlt-1/PIGF remains inconclusive or with a reduced prognostic value compared to our data [36,37]. One explanation for this observation might be the inconsistency in the reported outcome data; therefore, we decided to define APO according to the recently published Delphi consensus by Duffy et al. [29]. Another reason might be the focus on special cohorts, such as twin pregnancies [38]. Our results suggest that it might be necessary to investigate obese women as an independent subgroup to avoid a distorted result.

The main finding of our study is that APO can be predicted by sFlt-1/PIGF, not only in the group of normal and overweight women, but also in women with obesity, considering the decreasing prognostic value. This should be taken into account, especially if sFlt-1/PIGF levels are low. In contrast to normal and overweight women [39], obese women are at a higher risk of developing APO, even with sFlt-1/PIGF-levels < 38 and, therefore, these women might be in need of intensified clinical surveillance.

It is unclear why sFlt-1 levels are lower in obese women. In a Finnish case-control study, including 1450 women, sFlt-1 levels in pre-eclamptic obese women were significantly lower compared to women with normal BMI, whereas sFlt-1 levels in the first trimester did not differ [22]. In line with our own results, Suwaki et al. reported an inverse correlation of sFlt-1 and BMI in pre-eclamptic women [40], but no difference in the angiogenic profile of normotensive women. Lobmaier et al. could not find a significant influence of BMI on sFlt-1/PlGF levels, considering that the study group showed a relatively low BMI (22.5 (20.5–25.6) kg/m^2^) [20]. The profile of sFlt-1/PIGF in obese women without PE remains controversial, as some studies report a higher level of sFlt-1 and PIGF, as well as the opposite [41,42]. A possible explanation might be the above-mentioned “low BMI” study cohorts. Nevertheless, obese women affected by AMO, APO or PE show elevated levels of sFlt-1/PIGF compared to obese women without further complications [43].

Increased plasma volume, as a confounder, might, at least, contribute to our finding of a lower sFlt-1 level, as animal studies rather implicate a higher sFlt-1 release in obese pregnancies [44,45]. On the other hand, TNF-α, a proinflammatory factor elevated in obese women, decreases the sFlt-1 expression in fat tissue [46]. Another reason might be the higher mass of extracellular matrix in fat tissue containing heparin sulfate proteoglycans, which leads to sequestration of sFlt-1 from blood circulation [47]. However, PIGF concentrations were not different in obese and normal-weight women, which is in line with other studies [40]. Therefore, further research needs to be conducted to analyze the role of adipose tissue in the processing of angiogenic factors in PE.

The ratio of sFlt-1 and PIGF was not only a predictor of APO, but also of AMO, although its prognostic value is limited due to the low incidence of AMO in our study cohort. HELLP syndrome, as well as acute kidney failure, were more common in normal-weight women and other events were rare. This finding was unexpected, as obesity itself is a risk factor for HELLP syndrome or other pregnancy complications [48]. One explanation for this finding might be that women with the risk factor of obesity are carefully observed during pregnancy and, therefore, AMO might be prevented in some cases. Rana et al. demonstrated that pre-eclamptic women with a normal angiogenic profile were more likely to be obese and AMO was rare, except for iatrogenic preterm delivery [49]. Therefore, there might be a subgroup of obese women diagnosed with only mild symptoms of PE in the late-onset group, as obesity especially increases the risk of late-onset PE [50].

The limitations of our study were the retrospective design and a relatively small sample size. Furthermore, data on weight gain during pregnancy were not available for all women. We used the levels of sFlt-1/PIGF at the time of diagnosis of PE, but another measurement prior to delivery might give further and more detailed information about the angiogenic profile in PE women. Further, clinicians were aware of the test results. Therefore, sFlt-1/PIGF might have influenced the decision making, especially regarding the timing of delivery. On the one hand, this might have prolonged pregnancies if a low sFlt-1/PIGF was measured and helped to prevent APO. On the other hand, it might have led to an iatrogenic delivery if high serum levels were detected, especially if the mother was severely affected by PE and, therefore, might have prevented AMO. An impact of sFlt-1/PIGF in the decision-making process regarding further therapy of women with PE was described before [51]. Pre-pregnancy BMI was used for the statistical analysis; however, weight gain during pregnancy might influence our results. Interestingly, mean weight gain during pregnancy was lower in obese women. These results might be biased, as severe PE is often accompanied by excessive oedema. Another effect might be the diatetic counselling all obese women received during pregnancy.

## 5. Conclusions

In conclusion, we could demonstrate that sFlt-1/PIGF is a helpful tool for APO prediction in normal and overweight, but also in obese women. However, for decisions on the timing of delivery and perinatal management, it should be taken into account that the performance is not as precise in obese women as it is in normal-weight women. Clinicians should be aware that normal sFlt-1/PIGF might not rule out APO and, therefore, obese women with PE are a subgroup of patients who should be managed carefully.

## Figures and Tables

**Table 1:** Baseline characteristics.

Baseline Characteristics	BMI < 25 kg/m^2^ *n* = 84	BMI > 25–29.9 kg/m^2^ *n* = 29	BMI ≥ 30 kg/m^2^ *n* = 28	*p*-Value
Age at diagnosis	33.5 (±5.4)	34.1 (±5.7)	33.2 (±4.6)	0.608
BMI	21.2 (±1.8)	27.3 (±1.5)	34.1 (±3.9)	<0.001 ***
Weight gain during pregnancy (in kg)	13.5 (±6.1)	12.6 (±5.4)	10.9 (±7.0)	0.216
Gestational age at diagnosis	34.8 (±3.8)	34.8 (±3.9)	34.5 (±3.3)	0.680
sFlt-1/PIGF level at diagnosis	125.1 (IQR: 64.4–213.1)	116.0 (IQR: 45.3–233.0)	99.30 (IQR: 30.36–187.0)	0.275
PIGF level at diagnosis	93.9 (IQR: 54.6–93.9)	86.3 (IQR: 46.1–130.4)	89.35 (IQR: 51.0–130.9)	0.810
sFlt-1 level at diagnosis	12,024.8 (IQR: 7472.0–13,544.8)	8756.0 (IQR: 5708.5–13,040.0)	7014.0 (IQR: 3668.6–11,347.3)	0.003 **
Gestational age at delivery	35.5 (±3.3)	35.7 (±3.9)	35.9 (±3.0)	0.684
Nullipara	57 (68%)	17 (58%)	17 (61%)	0.396
ASS prophylaxis	10 (12%)	11 (38%)	9 (32%)	0.005 *
Chronic hypertension	8 (10%)	3 (10%)	3 (11%)	0.844
Early-onset PE/HELLP	24/84 (29%)	10/28 (36%)	10/29 (35%)	0.430
Late-onset PE/HELLP	60 (71%)	19 (66%)	17 (61%)	0.272
RDS Prophylaxis	27 (32%)	8 (28%)	11 (39%)	0.637
Status after PE/HELLP/FGR	16 (19%)	9 (31%)	8 (29%)	0.205
Magnesium sulfate prophylaxis	44 (52%)	16 (55%)	15 (54%)	0.868

BMI body mass index, sFlt-1 soluble fms-like tyrosine kinase-1, PIGF placental growth factor, ASS acetylsalicylic acid, PE pre-eclampsia, RDS respiratory distress syndrome, FGR fetal growth restriction. Data are presented as *n* (%) or mean ± SD or median and interquartile range. A *p*-value < 0.05 was considered as statistically significant (* *p* < 0.05; ** *p* < 0.005, *** *p* < 0.001).

**Table 2:** Perinatal outcome.

Perinatal Outcome	BMI < 25*n* = 84	BMI > 25–29.9 kg/m^2^ *n* = 29	BMI ≥ 30 kg/m^2^*n* = 28	*p*-Value
APO	46/84 (55%)	12/29 (41%)	11/28 (39%)	0.110
Respiratory support	27/84 (33%)	8/29 (28%)	7/28 (25%)	0.421
Admission to NICU	34/84 (41%)	10/29 (35%)	11/28 (39%)	0.764
Birthweight (g)	2243.2 (±48.4)	2422.1 (±932.5)	2456.3 (±841.3)	0.404
Birthweight < 3. centile	9/84 (11%)	2/29 (7%)	2/28 (7%)	0.751
Neonatal mortality	0/84 (0%)	0/29 (0%)	1/28 (4%)	0.080
Fetal growth restriction	24/84 (29%)	7/29 (24%)	5/28 (18%)	0.257
Seizures	1/84 (1%)	2/29 (7%)	0/28 (0%)	0.167
AMO	29/84 (35%)	3/29 (10%)	4/28 (14%)	0.010 *
Early-onset HELLP	8/84 (10%)	0/29 (0%)	2/28 (7%)	0.406
Late-onset HELLP	7/84 (8%)	1/29 (3%)	1/28 (4%)	0.297
Postpartum haemorrhage	1/84 (1%)	0/29 (0%)	1/28 (4%)	0.005 *
Eclampsia	0/84 (0%)	1/29 (3%)	0/28 (0%)	0.619
Acute kidney injury	12/84 (14%)	2/29 (7%)	1/28 (4%)	0.085
Abruption	3/84 (4%)	0/29 (0%)	0/28 (0%)	0.187

BMI body mass index, APO adverse perinatal outcome, NICU neonatal intensive care unit, AMO adverse maternal outcome. Data are presented as *n* (%) or mean ± SD or median and interquartile range. A *p*-value < 0.05 was considered as statistically significant (* *p* < 0.05).

## Data Availability

Not applicable.
